# Deactivation of default mode network during touch

**DOI:** 10.1038/s41598-018-37597-1

**Published:** 2019-02-04

**Authors:** Timmy Strauss, Robin Kämpe, J. Paul Hamilton, Hakan Olausson, Fabian Rottstädt, Claudia Raue, Ilona Croy

**Affiliations:** 1 Department of Psychosomatic Medicine and Psychotherapy, Technical University Dresden, Dresden, Germany; 2 Center for Social and Affective Neuroscience, Department of Clinical and Experimental Medicine, Linköping University, Linköping, Sweden; 3 Department of Neuroradiology, Medizinische Fakultät Carl Gustav Carus, Technische, Universität Dresden, Fetscherstr. 74, 01307 Dresden, Germany

## Abstract

Interpersonal touch possesses a strong affective component, which immediately evokes attention. The neural processing of such touch is moderated by specialized C-tactile nerve fibers in the periphery and results in central activation of somatosensory areas as well as regions involved in social processing, such as the superior temporal gyrus (STG). In the present functional neuroimaging investigation, we tested the hypothesis that the attention grasping effect of interpersonal touch as compared to impersonal touch is reflected in a more-pronounced deactivation of the default mode network (DMN). Using functional magnetic resonance imaging, we investigated the neural processing of interpersonal relative to impersonal touch conditions that were furthermore modulated by stroking velocity in order to target c-tactile nerve fibers to a different extent. A sample of 30 healthy participants (19 women, mean age 40.5 years) was investigated. In the impersonal touch, participants were stroked with a brush on the forearm. In the interpersonal touch condition, the experimenter performed the stroking with the palm of his hand. Interpersonal touch was rated as more pleasant and intense than impersonal touch and led to a stronger blood oxygen level dependent (BOLD) signal increase in the somatosensory cortex SII extending to the superior temporal cortex. Over all touch conditions, this activation was coupled in time to the deactivation of prominent nodes of the DMN. Although deactivation of the DMN was most pronounced for interpersonal touch conditions, the direct comparison did not show significant differences in DMN deactivation between interpersonal and impersonal touch or between different stroking velocities. We therefore conclude that all applied touch conditions deactivate the DMN and the strong connection to areas which code the contextual and social characteristics of affective touch may explain the attention grasping effect of touch.

## Introduction

As a central aspect of human interaction, affective touch makes critical contributions to important social experiences such as the hug from a friend, the endearment of a lover, or the comforting caress of a mother. Affective touch, in contrast to pure discriminative touch, creates a hedonic experience that evokes emotions. Hence, even in the absence of language, different emotions can be transported via touch^1^. Much research has however focused around the simple experience of affective touch being pleasant or unpleasant and different velocities of stroking have been used to modulate the affective touch perception^2^. Naturally, context plays a major role in the perception and processing of such touch^3,4^.

In the peripheral nervous system, certain touch stimulations are processed in a way that facilitates their perception as “affective”^2^. Light and slow stroking stimulations target c-tactile afferents; a group of nerve fibers that constitute a subgroup of unmyelinated c-fibers and that innervate human hairy skin. These fibers react selectively to slow stroking stimulation of about 1 to 10 cm/s and exhibit a reduced firing frequency when stroking is performed with higher or lower velocities^5^. Increased reactivity of unmyelinated c-fibers is strongly associated with participants’ pleasantness ratings of the stimulation^5^. Indeed, work across laboratories has confirmed that c-tactile optimal touch is, on average, rated as more pleasant than c-tactile suboptimal touch^2,5–8^. C-tactile fibers are also sensitive to temperature, with the highest firing frequency occurring when slow stroking stimulation is presented at about 32 °C – a temperature which resembles skin-to-skin contact^9^. Given these characteristics, it has been proposed that c-tactile fibers are tuned to social, interpersonal, touch^10,2^. Interestingly, stimulation of these fibers is not only perceived as pleasant, it also has a calming effect on the autonomic nervous system, as reflected in reduced heart rate^11^ and enhanced heart rate variability^12^.

The neural processing of c-tactile, affective touch involves activation of the primary somatosensory cortex (SI), secondary somatosensory cortex (SII), the superior temporal gyrus (STG), the posterior insular cortex, the orbitofrontal gyrus as well as the anterior cingulate cortex (e.g.^13–17^). The activation of primary^3^ and secondary^4^ somatosensory cortex by c-tactile optimal stimulation is moderated by contextual information during touch presentation; moreover, individual perception of touch pleasantness correlates with evoked activity in the superior temporal sulcus^13^ and the anterior cingulate cortex^17^. In the aforementioned studies, affective touch was applied by c-tactile optimal stimulation of 3 cm/s, performed with a brush on participants’ forearm or leg. This approach, however, is somewhat limited as it neglects the interpersonal aspect of affective touch that occurs, for example, when stroking is performed with the palm of the hand.

Given that touch signals the presence of others, it may initiate a shift in brain processing associated with an attention to the outer world. We propose that this shift is reflected in altered default mode network (DMN) responding. The DMN is a large scale brain network which is typically deactivated by the shifts towards external attention, represented by social cues^18,19^, focused attention^20^ and externally oriented cognitive tasks^21^. The DMN comprises a few primary nodes. The ventral medial prefrontal cortex (VMPFC) is a neural hub in which sensory information is gathered, weighted and conveyed to the amygdala and the hypothalamus. Another node, the dorsal medial prefrontal cortex (DMPFC), is located in close proximity to the VMPFC and is involved in self-referential judgments. The posterior cingulate cortex (PCC), a third node, is involved in the recollection of prior experiences, as are the inferior parietal lobule (IPL), the lateral temporal cortex (LTC) and the hippocampal and parahippocampal formation (HF)^22^.

We aimed in the present investigation to examine the neural processing of affective and, in particular, interpersonal touch. We expected that the interpersonal component of the touch as well as the degree of c-tactile fiber stimulation would modulate the affective perception and processing of touch. Interpersonal touch was operationalized by stroking performed with a brush vs with the experimenters’ hand. C-tactile targeted touch was operationalized by different velocities of stroking (3 cm/s = c-tactile targeted touch; 30 cm/s = non-c-tactile targeted touch). To address our research question, we conducted functional magnetic resonance imaging (fMRI) under four experimental conditions: (1) c-tactile targeted, interpersonal touch, (2) c-tactile-targeted, impersonal touch, (3) non-c-tactile targeted, interpersonal touch and (4) non-c-tactile targeted, impersonal touch.

We hypothesized that these touch conditions can be ranked according to their pleasantness ratings with non-c-tactile targeted, impersonal touch being most neutral and c-tactile targeted, interpersonal touch being rated as most pleasant. We assumed further, that all touch conditions would result in an activation of tactile eloquent areas and in a deactivation of the DMN nodes. These effects were assumed to be most pronounced in the interpersonal touch conditions. We furthermore aimed to examine the neural temporal coupling between tactile eloquent areas and the DMN in affective touch conditions.

## Materials and Methods

### Participants

Thirty-one right-handed participants (19 women, mean age 40.5 +− 2.8 years SD) were examined for this experimental study. As depression affects DMN activity^23^, all participants were screened for symptoms of this disorder with the well-validated Beck Depression Inventory (BDI)^24^; no participant scored in the range of moderate-to-severe depression (cut off 14 points). Furthermore, all participants were screened for autistic traits using the Autism-spectrum quotient (AQ)^25^, given that autistic traits are related to altered c-tactile perception and processing^26,27^. None of the participants scored above the recommended AQ cutoff of 34 points. One of the participants was later excluded due to technical problems during data acquisition.

The study followed the guidelines of the Declaration of Helsinki on Biomedical Research Involving Human Subjects and was approved by the Ethics Committee of the TU Dresden (EK 533122015).

### Procedure

After providing informed consent and prior to scanning, participants completed the Positive and Negative Affect Schedule (PANAS^28^ for assessment of current mood (group mean of positive emotions: 38.03 +− 1.1 SD/negative emotions: 16.3 +− 0.78 SD).

### fMRI acquisition

Functional magnetic resonance imaging data were acquired on a 3 Tesla MR scanner (Trio; Siemens Medical, Erlangen, Germany), equipped with an 8-channel head coil, using a protocol with a T2*-weighted echo-planar imaging (EPI) sequence (TR = 3 s, TE 51 ms, flip angle 86° (optimized to be Ernst angle), in-plane resolution 3.6 × 3.6 mm^2^, slice thickness = 6 mm, no slice gap, angled with the AC-PC line). Three dummy volumes were acquired before each scan to allow the spin system to reach steady-state longitudinal magnetization and reduce possible effects of partial saturation. A high resolution T1 sequence (3D IR/GR sequence: TR = 3 s, 0.7 × 1 mm^2^ in-plane resolution) was added for precise anatomical mapping of functional neuroimaging data as well as exclusion of participants with potential brain pathology.

### Touch presentation

Stroking was performed on participants’ left forearms in four randomized runs: (a) interpersonal touch (performed with a human hand) with c-tactile targeted velocity of 3 cm/s, (b) interpersonal touch with non-c-tactile targeted velocity of 30 cm/s, (c) impersonal touch (performed with a brush) with c-tactile-targeted velocity of 3 cm/s and (d) impersonal touch with non-c-tactile-targeted velocity of 30 cm/s.

All stroking conditions were performed manually by the experimenter (TS) who stroked the participants on their left dorsal forearm over a distance of 10 cm from proximal to distal direction. A computer program guided the experimenter. During the interpersonal touch conditions, the experimenter stroked with his palm, during the impersonal touch conditions, he stroked with a 50 mm wide flat, soft watercolor brush made of fine soft goat hair. A potential difference of temperature between both types of stimulation may affect the reactivity of c-tactile fibers^29^. Therefore, the experimenter made sure that his hand temperature was similar to the forearm skin temperature before each run. If his hands were too cold, he rubbed them until there was no perceptual temperature difference between the experimenter’s hand and the subject’s forearm. Presenting constant force and velocity of stroking was extensively practiced by the experimenter prior to testing. For further details on stroking preparation, especially for control of force and velocity, please see the supplement to this article. Given our use of computer-guided velocity, we assume that the experimenter’s manual touch administration in the impersonal touch condition is perceived similar to robot-guided administration of tactile stimuli as shown previously^30^.

**Figure 1:**
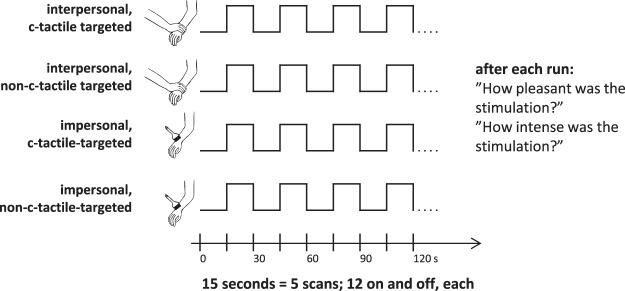
A visual schematic of our functional magnetic resonance imaging (fMRI)-design. Each participant underwent four different stroking conditions during fMRI scanning in a randomized order. In every condition, stroking touch was applied in a block design with 12 repetitions of On and Off. After each of the conditions, the participants verbally assessed both the pleasantness and intensity of the touch percept. Interpersonal touch: touch applied by a human hand; impersonal touch: touch applied with a brush; c-tactile targeted: velocity of 3 cm/s; non-c-tactile targeted: velocity of 30 cm/s.

Each run consisted of 12 stimulation periods (15 seconds of stimulation followed by 15 seconds of non-stimulation), summing to 6 minutes of scanning per run (see Fig. 1). In the 15 seconds time interval, 4 strokes were performed during the c-tactile targeted stimulation and 40 strokes during the non-c-tactile targeted conditions. The experimenter initiated and discontinued touch for a given stimulation period by observing signals for the beginning and end of each block that were displayed on a monitor, which was only visible to the experimenter. Our participants were instructed to close their eyes during scanning procedure and this behavior was monitored.

After each run, participants were asked to verbally assess the pleasantness and the intensity of the stroking experience on a visual analogue scale (pleasantness: −5 [very unpleasant] to +5 [very pleasant]; intensity: 0 [not intense at all] to 10 [very intense]). As we detail below, this assessment guided the MRI analysis in an additional post-hoc analysis. So that pleasantness and intensity ratings from the subject did not inadvertently influence the stroking procedure carried out by the experimenter, a set of safeguards was put in place. The experimenter received no visual feedback from the patients. In addition, behavioral ratings provided by the participants were given directly to a technical assistant and the experimenter was shielded auditory from the participant, which blocked the experimenter from verbal feedback.

### Analysis of Behavioral Data

Intensity and pleasantness ratings were separately analyzed using SPSS v. 22 with a 2 × 2 repeated measurement ANOVA. The normality assumption was not fulfilled for most of the ratings. Despite the skewed distribution, we used a repeated measurements ANOVA, which is robust against violations of the normality assumption for sample sizes of n > 25. Two within-subject variables were included: interpersonal character of stroking (interpersonal vs impersonal) and c-tactile-targeted character of stroking (3 vs 30 cm/s stroking velocity) and both main effects and the interaction were modeled. The optimal linear contrast of this ANOVA yielded in the “touch pleasantness weighted” contrast.

### fMRI analysis

#### Preprocessing and single subject analysis

The analysis of fMRI time-series data was implemented in a standard general linear model approach in SPM12 (Statistical Parametric Mapping; Welcome Department of Imaging Neuroscience, in the Institute of Neurology at University College London [UCL], UK) incorporated in Matlab (Matlab 9.1, The MathWorks IncS., Natick, MA). FMRI data preprocessing started with realignment of functional acquisitions relative to a reference acquisition using a 2^nd^ degree B-spline method. Next, the data were high-pass filtered using cutoff at a period of 128 s, and then normalized using the segmentation procedure implemented in SPM 12 with affine registration to the International Consortium for Brain Mapping (ICBM) space template (Montreal Neurological Institute [MNI] space). Finally, we performed bias regularization (0.0001), and spatial smoothing with a Gaussian kernel of 6 × 6 × 6 mm^3^ Full Width at Half Maximum (FWHM).

For the first-level multiple-regression analysis of the fMRI time-series data, a boxcar covariate reflecting stimulus on-off cycles convolved with SPM’s canonical hemodynamic response function was used. Motions-based noise regressors (which were very limited for each participant and run; <2 mm cumulative translation and 1° cumulative rotation) were included in our analysis.

#### Group level analysis – activation

In order to *examine the activation of tactile eloquent areas*, a full factorial design was implemented with the factors of interpersonal (interpersonal vs impersonal) and c-tactile targeted (3 vs 30 cm/s stroking velocity), using the individual task vs baseline contrasts for each touch condition.

In order to compare the averaged response per condition to the baseline, four t-statistics were calculated, one per touch condition. Afterwards, the interpersonal touch conditions were contrasted to the impersonal ones and c-tactile targeted to the non-c-tactile targeted ones, using t-contrasts.

With the aim of grouping all different conditions into one t-map, which includes all neural responses in a way that best reflects the pleasantness ratings, we computed a weighted pleasantness contrast. This “touch pleasantness weighted” contrast was guided by the *post-hoc* observation in the behavioral data and weighted the four touch conditions according to the ranking of their pleasantness ratings (impersonal, c-tactile targeted: 1; interpersonal, c-tactile targeted: 0.75; impersonal, non-c-tactile targeted: 0.5; interpersonal, non-c-tactile targeted: 0.25). As for the individual conditions, this “touch pleasantness weighted” contrast was tested against baseline. Resulting activation clusters were used as regions of interest (ROI) for further analysis. The averaged region-wise beta values were extracted from those clusters, which highly overlapped with the clusters observed in each of the four touch conditions using Marsbar^31^. This data was entered into an automatic linear model analyzed (SPSS 24) with the predictors “pleasantness”, “intensity”, and “condition”. This was done in order to test the moderating effect of pleasantness and intensity on the neural differences, observed between the conditions.

For all SPM analyses, a whole brain approach was used with the statistical threshold of α =0.05, family-wise error (FWE) corrected at the peak level in order to correct false-positive findings as recommended by Eklund *et al*.^32^.

Found regions were anatomically defined by the anatomy toolbox implemented in SPM12^33^.

#### Group level analysis - deactivation

In the next step, we identified regions showing a *deactivation of the Default Mode Network*. Therefore, the same full factorial design matrix was used as reported above and for each touch condition, contrasts were set reversely in order to obtain deactivations as compared to baseline. The interpersonal vs impersonal and c-tactile targeted vs non-c-tactile targeted touch conditions were tested against each other. A whole brain approach was used with the statistical threshold of α = 0.05, family-wise error (FWE) corrected. In order to specifically test the hypothesis that affective touch leads to a deactivation of the DMN, a focused analysis was performed with the DMN nodes as ROIs (see^34^). However, as^34^ show a high overlap of areas included in the vmPFC and dmPFC, both regions were joined into a single “mPFC” ROI in our analysis (Table 1). The ROIs were extracted via WFU pickatlas^35^ and were anatomically defined by their respective Brodmann areas using the anatomy toolbox^33^. Statistical threshold was again set to =0.05, family-wise error (FWE) corrected.

**Table 1:** Nodes of Default Mode Network (modified from Buckner *et al*.^34^).

DMN node	Abbreviation	Included brain areas [Brodmann areas]
Medial prefrontal cortex	mPFC	24, 10 m/10r/10p, 32ac, 9
Posterior cingulate/retrosplenial cortex	PCC/Rsp	29/30, 23/31
Inferior parietal lobule	IPL	39, 40
Lateral temporal cortex	LTC	21
Hippocampal formation	HF+	Hippocampus proper, EC, PH

The authors show a high overlap of areas included in the ventral medial prefrontal cortex and the dorsal medial prefrontal cortex. That is why we joined both regions into a single “medial prefrontal cortex” (mPFC) ROI in our analysis.

In a post hoc analysis, we also examined the whole brain deactivation and the ROI deactivation for the “touch pleasantness weighted” contrast.

#### PPI Analysis

Our observation of the deactivation of the DMN by affective touch was followed by investigating the *temporal correlations between the regions activated by the touch conditions and the DMN*. Therefore, a psychophysical interaction (PPI) was performed with the two seeds that were determined as activation clusters from the “touch pleasantness weighted” contrast: the SI and the SII including the STG.

To run the PPI analysis, the beta values were implemented into AFNI (Analysis of Functional NeuroImages, version 17^36^) and we set the presented regions of interests as masks, called “activation”.

The data were despiked and detrended using polynomial functions up to the third degree (determined by run length) to model any potential drift over time. The averaged time series were extracted within the mask “activation” containing SII/STG and SI, resulting in two time series per subject. PPI regressors were generated for each individual subject by first creating a hemodynamic response function using the AFNI waver function. The seed (SII/STG and SI) time series were deconvolved from left to right with the generated hemodynamic response (HDR) function to obtain the underlying neural response. From the neural response function four interaction files were extracted by multiplying the neural response function with the block-functions of the four touch conditions. To translate the interaction functions back to the hemodynamic domain these functions were convolved with the HDR function from left to right giving us four different PPI-regressor per condition and region (total: 8 per subject).

To obtain voxelwise PPI coefficients, we used a general linear model regression including six regressors for motion (x, y, z, roll, pitch, and yaw), the time series from the seed region, the four block-signals (HRF-convolved) of the four touch conditions and the four PPI interaction regressors.

We then converted the PPI coefficient maps into Nifti-format and proceeded to analyze them in SPM 12. The full factorial second-level analysis included these PPI coefficient maps and the interpersonal touch and impersonal touch as well as the c-tactile targeted and non-c-tactile targeted conditions were contrasted to each other, using t–statistics.

## Results

### Behavioral Results

**Figure 2:**
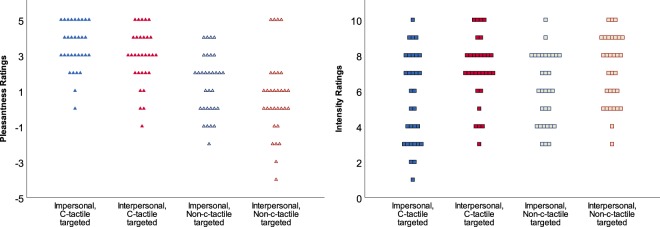
Behavioral data. Left panel: Pleasantness ratings of the different touch conditions show a linear decreasing trend of pleasantness ratings with c-tactile-targeted conditions being rated as more pleasant than the non-c-tactile-targeted touch conditions (p < 0.001) and impersonal touch conditions being rated as more pleasant than interpersonal touch (p = 0.017). Right panel: Intensity ratings show that interpersonal touch was rated as more intense than impersonal touch (p < 0.001).

The touch conditions differed significantly in the affective touch perception as operationalized by pleasantness ratings. C-tactile-targeted conditions were consistently rated as more *pleasant* than the non-c-tactile-targeted touch conditions (F[1, 29] = 50.1, p < 0.001; η² = 0.63, see Fig. 2). Furthermore, and unexpectedly, the impersonal touch conditions were rated as more pleasant than the interpersonal ones (F[1, 29] = 5.84, p = 0.022; η² = 0.17). There was no significant interaction between the interpersonal touch and c-tactile-targeted conditions (p = 1.0).

The touch conditions could be ranked according to pleasantness ratings with a linear decreasing effect (F[1, 29] = 52.2, p < 0.001; η² = 0.64): (1) impersonal, c-tactile targeted touch, (2) interpersonal, c-tactile targeted touch, (3) impersonal, non-c-tactile targeted and (4) interpersonal, non-c-tactile targeted touch. We called this order “touch pleasantness weighted”.

Interpersonal touch conditions were rated as more *intense* than the impersonal touch conditions (F[1, 29] = 32.8, p < 0.001; η² = 0.53). No significant effect was observed for the comparison between c-tactile-targeted (slow stroking) and non-c-tactile-targeted (fast stroking) conditions (p = 0.25) or for the interaction of the conditions (p = 0.11).

### BOLD response to affective touch

#### Activation of tactile eloquent areas

**Figure 3:**
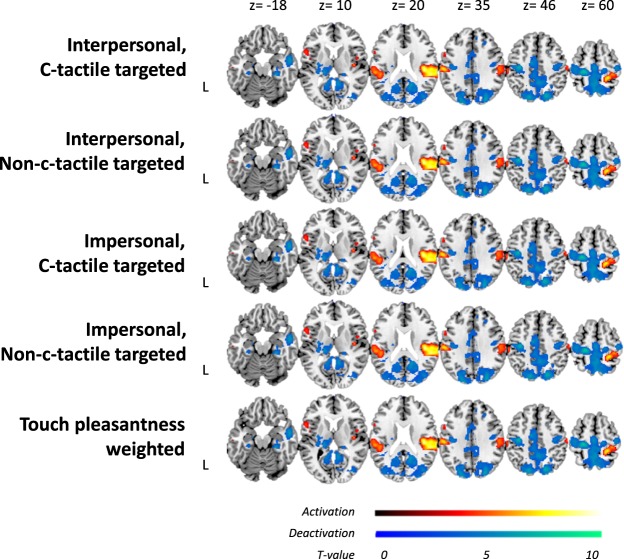
BOLD signal in relation to baseline. The enhanced and reduced activations as compared to baseline are displayed in red or blue color, respectively, for each of the interpersonal and impersonal touch conditions as well as for the “touch pleasantness weighted” contrast. Data is presented on a template provided by Marsbar with an uncorrected threshold of p < 0.001 for visualization purpose. Each of the touch conditions activated the secondary somatosensory cortex SII extending to the STG and the primary somatosensory cortex SI. This effect was stronger for interpersonal than for impersonal touch conditions. Moreover, we identified reduced BOLD response within the DMN in each of the four touch conditions.

All of the four touch conditions led to a strong blood oxygen level dependent (BOLD) signal increase in the secondary somatosensory cortex which extended to the superior temporal gyrus and in the primary somatosensory cortex (see Table 2, Fig. 3 and Supplementary Figures for inverted full hemisphere brain images). In line with our hypothesis, these activations were more pronounced in the interpersonal compared to impersonal touch conditions (Table 2). This is reflected in a 58% stronger T-value in interpersonal as compared to impersonal touch conditions in the SII/STG region. Furthermore, c-tactile targeted touch led to an enhanced activation in SI as compared to non-c-tactile targeted touch (Table 2). In addition, the c-tactile stimulation led to enhanced activation in previously reported areas, such as the posterior insula and the orbitofrontal cortex, but those responses missed the required statistical threshold.

**Table 2:** Comparison of neural activations between touch condition vs baseline and between specific touch conditions.

	Condition	Cluster size	T value	MNI coordinates x y z			FWE corrected p value: peak level
	**Interpersonal Touch, c-tactile targeted**						
Activation in touch conditions vs Baseline	R SII extending to Superior Temporal Gyrus	1978	12.31	49	−28	26	<0.001
	L SII extending to Superior Temporal Gyrus	1058	7.31	−57	−20	36	<0.001
	R Postcentral Gyrus (Brodmann Area 2/3b/4a/4p)	778	7.46	25	−38	60	<0.001
	**Interpersonal Touch, non-c-tactile targeted**						
	R SII extending to Superior Temporal Gyrus	1663	12.76	47	−30	26	<0.001
	L SII extending to Superior Temporal Gyrus	1012	9.00	−47	−36	24	<0.001
	R Postcentral Gyrus, Brodmann Area 2/3b/4a/4p	817	10.50	33	−30	62	<0.001
	**Impersonal Touch, c-tactile targeted**						
	R SII extending to Superior Temporal Gyrus	815	6.97	49	−28	26	<0.001
	L SII extending to Superior Temporal Gyrus	664	6.75	−55	−22	34	<0.001
	**Impersonal Touch, non-c-tactile targeted**						
	R SII extending to Superior Temporal Gyrus	654	7.16	49	−28	26	<0.001
	R Thalamus: temporal	579	5.23	−21	−44	14	<0.001
	**Touch pleasantness weighted**						
	R SII extending to Superior Temporal Gyrus	965	13.13	49	−28	26	<0.001
	L SII extending to Superior Temporal Gyrus	576	8.44	−59	−20	40	<0.001
	R Postcentral Gyrus	78	6.23	21	−42	62	<0.001
Compared Activations	**Interpersonal Touch > Impersonal Touch**						
	R Brodmann Area 4a/3b/2, Postcentral Gyrus	984	7.68	31	−26	60	<0.001
	R SII	391	5.14	41	−24	20	<0.001
	**Impersonal Touch > Interpersonal Touch**						
	No suprathreshold activation, pFWE < 0.05						
	**C-tactile targeted Touch > Non-c-tactile targeted Touch**						
	R Brodmann Area 1/2/3b, Postcentral Gyrus	435	5.31	53	−20	34	<0.001
	**Non-c-tactile targeted Touch > C-tactile targeted Touch**						
	L Thalamus temporal, Cerebellum (Lobule IV-V)	492	5.07	3	−38	−2	<0.001
	R Brodmann Area 4a/p, Superior Parietal Lobe	376	5.02	29	−28	60	<0.001

Data was extracted from a whole-brain analysis and only activations with peak-level FWE-corrected p-value < 0.05 are reported. Regions were anatomically defined with the anatomy toolbox^33^.

For a post-hoc inspection of our data we used the “touch pleasantness weighted” contrast that weighted the touch conditions according to their pleasantness ratings. As compared to baseline, this contrast led to the same activation within the SI and the SII/STG cluster, with a high overlap to each of the individual conditions (compare Fig. 3 and Table 2). The extracted beta values of the SII/STG cluster was increased in the interpersonal as compared to the impersonal touch conditions (F[1, 29] = 7.1, p = 0.012, η² = 0.19), while there was no effect of c-tactile targeted conditions (p = 0.46) and no interaction effect (p = 0.96). An automatic linear modeling procedure with the predictors “pleasantness”, “intensity”, and “condition” revealed that these differences were best explained by intensity ratings (F(1, 123] = 9.5, p = 0.003, predictor importance 0.749). For the SI activation cluster again, interpersonal touch conditions were related to enhanced BOLD signal compared to the impersonal ones (F[1, 29] = 15.3, p < 0.001, η² = 0.338), while there was no effect of c-tactile targeted conditions (p = 0.48) or the c-tactile targeted-by-interpersonal interaction (p = 0.26). The automatic linear modeling showed that only “condition”, but not “intensity” or “pleasantness” was related to SI activation (F[1, 123] = 7.5, p = 0.007).

#### Deactivation of Default Mode Network

We identified *reduced BOLD response* within the DMN in each of the four touch conditions (Fig. 3 and Table 3, compare also Supplementary Table S1 for whole brain deactivation). However, for the impersonal, non-c-tactile-targeted touch a more liberal threshold of p < 0.001, uncorrected, is needed in order to detect the effect. Although, the interpersonal touch conditions led to a more pronounced BOLD signal reduction in the DMN, the direct comparison between touch conditions showed no significant effect.

**Table 3:** Reduced BOLD signaling compared to baseline within the hypothesized ROIs of the default mode network in each condition and the “touch pleasantness weighted” contrast. Results are presented FWE-corrected.

Condition	DMN node	Cluster size	T value	MNI coordinates x y z			FWE corrected p value: peak level
**Interpersonal touch, C-tactile targeted**	**mPFC**						
	I	38	6.29	−11	−10	42	<0.001
	**PCC/Rsp**						
	I	5	5.50	−5	−20	50	<0.001
	**IPL**						
	No suprathreshold activation * activated with a more liberal threshold						
	**LTC**						
	I	9	5.94	55	−4	−18	<0.001
	II	7	6.05	57	−8	−6	<0.001
	**HF/PH**						
	I	49	6.65	25	−38	−10	<0.001
	II	8	5.68	29	−26	−22	<0.001
**Interpersonal touch, Non-c-tactile-targeted**	**mPFC**						
	I	6	5.32	−9	−8	42	<0.001
	**PCC/Rsp**						
	No suprathreshold activation * activated with a more liberal threshold						
	**IPL**						
	I	5	5.32	45	−74	8	<0.001
	**LTC**						
	No suprathreshold activation * activated with a more liberal threshold						
	**HF/PH**						
	I	30	6.01	29	−22	−22	<0.001
	II	5	5.73	23	−34	−14	<0.001
**Impersonal touch, C-tactile-targeted**	**mPFC**						
	No suprathreshold activation * activated with a more liberal threshold						
	**PCC/Rsp**						
	I	30	6.16	15	−62	26	<0.001
	II	15	5.10	−3	−42	44	<0.001
	**IPL**						
	No suprathreshold activation * activated with a more liberal threshold						
	**LTC**						
	No suprathreshold activation * activated with a more liberal threshold						
	**HF/PH**						
	I	12	5.27	29	−28	−8	<0.001
	II	8	5.15	29	−30	−18	<0.001
**Impersonal touch, Non-C-tactile-targeted**	**mPFC**						
	No suprathreshold activation * activated with a more liberal threshold						
	**PCC/Rsp**						
	No suprathreshold activation * activated with a more liberal threshold						
	**IPL**						
	No suprathreshold activation * activated with a more liberal threshold						
	**LTC**						
	No suprathreshold activation * activated with a more liberal threshold						
	**HF/PH**						
	No suprathreshold activation * activated with a more liberal threshold						
**Touch pleasantness weighted**	**mPFC**						
	I	174	7.86	−9	−8	42	<0.001
	II	43	6.00	3	4	40	<0.001
	**PCC/Rsp**						
	I	93	6.82	−3	−20	50	<0.001
	II	78	6.26	11	−60	22	<0.001
	III	58	6.50	−9	−46	6	<0.001
	IV	13	6.17	15	−34	44	<0.001
	V	2	5.78	25	−78	18	<0.001
	**IPL**						
	I	10	6.29	45	−74	8	<0.001
	II	10	5.66	−41	−76	24	
	III	5	5.81	43	−78	8	<0.001
	**LTC**						
	I	21	6.93	53	−6	−18	<0.001
	**HF/PH**						
	I	142	7.09	29	−30	−18	<0.001
	II	56	6.27	29	−30	−6	<0.001

DMN nodes were a priori-defined. In result, we identified reduced BOLD response within the DMN in each of the four touch conditions and the “touch pleasantness weighted” contrast (please note: for a sufficient deactivation of DMN in the impersonal, non-c-tactile targeted condition, a more liberal threshold of p < 0.001, uncorrected, needs to be applied). Labels I to VI are different clusters within the same anatomical ROI. Direct comparison between the four touch conditions revealed no significant effect.

mPFC = medial prefrontal cortex; PCC = posterior cingulate cortex; IPL = inferior parietal lobule; LTC = lateral temporal cortex; HF/PH = hippocampal and parahippocampal formation.

A *post-hoc analysis* showed that the “touch pleasantness weighted” contrast was associated with deactivation of each of the DMN nodes (Table 3). As power increased by combining all 4 conditions, the effect of DMN activity reduction was strongest in this contrast.

### Psychophysical Interaction in affective touch

**Figure 4:**
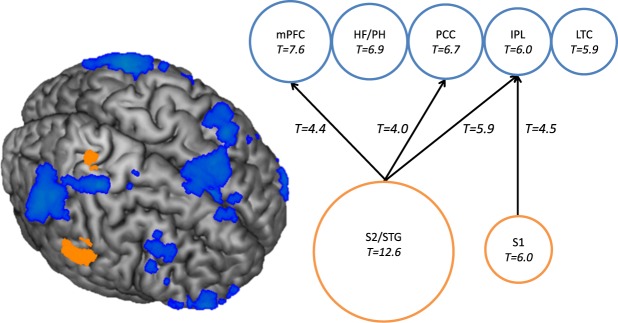
The psychophysical interaction analysis revealed a significant negative coupling between SII/STG and multiple areas of the default mode network during affective touch. Left side: Significant activation and deactivation is displayed on a standard template provided by MRIcron with Skin Threshold +30% and Search Depth of +12 mm. Right side: Coupling between SI and the default network nodes was weaker in general. The strength of coupling is displayed as T-values next to the arrows. The strength of activation (orange) in SII/STG and SI as well as the strength of deactivation in the default network nodes (blue) is also indicated by T-values, presented in the blobs. The size of the blobs furthermore codes the strength of BOLD signal change in the “touch pleasantness weighted” contrast compared to baseline. (mPFC = medial prefrontal cortex; HF/PH = hippocampal and parahippocampal formation; PCC = posterior cingulate cortex; IPL = inferior parietal lobule; LTC = lateral temporal cortex).

In order to investigate whether there were temporal correlations between tactile eloquent regions and the DMN, psychophysical interaction analyses were performed. The two activated clusters identified in the “touch pleasantness weighted” contrast served as seed regions. SII/STG activation correlated negatively with activation in mPFC, PCC and IPL regions of the DMN. The relation between SI and the DMN nodes was less pronounced and significantly observed for the IPL node of the DMN only (see Table 4 and Fig. 4).

**Table 4:** PPI-Analysis: Regions that are negatively correlated with SI and SII/STG activation.

Seed	DMN node	Cluster size	T value	MNI coordinates x y z			FWE corrected p value: peak level
**SII/STG**	**mPFC**						
	I	132	4.67	−51	48	0	0.04
	II	30	4.75	45	52	−8	0.01
	III	20	4.32	−5	22	46	0.03
	**PCC/Rsp**						
	I	7	3.98	3	−24	34	0.05
	**IPL**						
	I	378	5.89	−47	−56	54	<0.001
	II	340	5.10	51	−58	44	0.002
	**LTC**	No suprathreshold activation					
	**HF/PH**	No suprathreshold activation					
**SI**	**mPFC**	No suprathreshold activation					
	**PCC/Rsp**	No suprathreshold activation					
	**IPL**	No suprathreshold activation					
	I	178	4.30	−47	−56	54	0.02
	**LTC**	No suprathreshold activation					
	**HF/PH**	No suprathreshold activation					

α level was set to 0.05, FWE corrected. mPFC = medial prefrontal cortex; PCC = posterior cingulate cortex; IPL = inferior parietal lobule; LTC = lateral temporal cortex; HF/PH = hippocampal and parahippocampal formation.

Psychophysical interactions between SII/STG and DMN were however not significantly stronger in interpersonal vs impersonal touch or the reverse contrast and they were not significantly affected by the c-tactile-targeted character of touch.

## Discussion

In line with our hypothesis, neural activation was affected by our manipulations of touch. Consistent with the enhanced intensity perception of the interpersonal touch conditions, we observed a stronger BOLD signal increase of the tactile eloquent regions of SI and SII/STG in the interpersonal compared to the impersonal touch conditions. This is consistent with previous results showing that activation in the primary and in the secondary somatosensory areas is correlated to the intensity perception of touch^17,37^ and it is consistent with previous results showing that the intensity of SII reactivity is moderated by context effects. In this investigation a more or less pleasant odor was presented simultaneously to the touch presentation^4^. In another study, people were led to believe that the touch stimulation was performed by either a man or a woman and no effect on SII was reported, but on the SI^3^. Taken together the two aforementioned studies and our results suggest that the SII and potentially the STG may be tuned to the sensory integration of touch properties and to their interpersonal context.

In contrast to the findings we observed for the intensity ratings, the ratings of touch pleasantness tracked less well with interpersonal versus impersonal touch conditions, but strongly with the velocity of touch. C-tactile-targeted stroking velocities were experienced as substantially more pleasant than non-c-tactile-targeted ones. This latter result is consistent with previous findings (e.g.^5^), showing an enhanced preference of slow, c-tactile targeted over fast, non-c-tactile targeted touch. These behavioral effects of velocity of touch were, however, not reflected in the neural activation patterns.

Consistent with our hypothesis, a DMN BOLD response deactivation was found across all touch conditions. Deactivation of the DMN is observed following auditory or visual stimulation^38^; here, we extend this finding to the domain of affective touch. In contrast to our hypothesis, there was no significant effect of touch condition on the DMN deactivation, although the deactivation was more pronounced in the interpersonal as compared to the impersonal touch conditions. The psychophysical interaction analysis showed that the DMN deactivation was strongly related to the task dependent activation in the tactile eloquent areas, which was found irrespective of the touch condition applied. The SII and the STG were more strongly negative correlated to the DMN than SI. Consistent with this finding, it has been previously shown that the DMN interacts with the superior temporal region in social cognition tasks^39^. In particular, previous observations of a negative correlation between the PCC node of the DMN and the STG have been interpreted as indicators of social cognition^40^. This aligns not only with our result of negative coupling between STG and PCC, but also with our finding of enhanced STG activation in interpersonal than in impersonal touch. In previous research, the STG was correlated with the pleasantness of touch (as it is a function of the STG - Björnsdotter & Olausson^14^).

Activation of the SII/STG was also strongly negatively associated to the medial prefrontal nodes of the DMN in the present investigation. This is an intuitive finding, as the VMPFC node is seen as a neural hub, which gathers and disposes of sensory information^22^. Activations of the IPL as well as the PCC have previously been associated with the recollection of prior experiences^22^. The strong negative association of these regions to the SII/STG observed in our study, may reflect that assigning hedonic value to current touch experiences is mapped to previous experiences.

We assume that the enhanced intensity rating of the interpersonal touch conditions as compared to the impersonal touch conditions, reflects an inherent high salience of human touch. However this interpretation is limited by the following methodological specificities.

As the interpersonal touch condition was performed by the palm of the hand, it stimulated a larger skin surface on the participants forearm than impersonal touch, performed by a brush and there may have been subtle differences in applied force of stimulus presentation. We aimed to minimize this effect by practicing the stroking before the experiment; however, we did not monitor the force of stroking during the experiment. Due to this limitation, enhanced activation of tactile eloquent areas in interpersonal touch conditions may have been driven by methodological differences in stimulus presentation. Further studies with high controlled force and skin surface touched are warranted to clarify this question.

Another limitation relates to the touch conditions being rather pleasant which results in a roof effect with a limited variation of the pleasantness ratings. A larger variation of affective touch conditions - potentially also targeting the unpleasant side – is warranted in further studies.

In conclusion, different touch conditions resulted in cortical activation of tactile eloquent areas. This effect was especially pronounced in interpersonal touch conditions. Touch was furthermore associated with a strong deactivation of the DMN. This DMN activation decrease was coupled in time to the activation of secondary somatosensory cortex and to the STG – regions that are known to code the contextual effects of tactile stimulation.

## Supplementary information

Supplement

## Data Availability

The datasets generated during and/or analysed during the current study are available from the corresponding author on reasonable request.
