# Supplementary material for: Deactivation of default mode network during touch
Source: Sci Rep. 2019 Feb 4;9:1293. doi: 10.1038/s41598-018-37597-1 (PMC6361921; doi:10.1038/s41598-018-37597-1)
Supplement: Supplementary file 1 — Supplement [file 41598_2018_37597_MOESM1_ESM.pdf]

Supplement to

Deactivation of default mode network during touch

Strauss*, Timmy [a]; Kämpe, Robin [b]; Hamilton, J. Paul [b]; Olausson, Hakan [b]; Rottstädt, Fabian [a]; Raue, Claudia [c]; Croy, Ilona [a]

Affiliations

[a] Department of Psychosomatic Medicine and Psychotherapy, Technical University Dresden, Dresden, Germany

[b] Center for Social and Affective Neuroscience, Department of Clinical and Experimental Medicine, Linköping University, Sweden

[c] Department of Neuroradiology, Medizinische Fakultät Carl Gustav Carus,
Technische
Universität Dresden, Fetscherstr. 74, 01307 Dresden, Germany

***Corresponding author**

Cand. med. Timmy Strauss
University of Dresden Medical School
Department of Psychosomatic Medicine and Psychotherapy
Fetscherstraße 74
01307 Dresden
Germany
Mail: timmy.strauss@uniklinikum-dresden.de

Short title: **Deactivation of default mode network during touch**

Keywords: fMRI, touch, interpersonal, default mode network

Preparation of stroking prior to data acquisition

Preparation of stroking was done by stroking on a human forearm with a 10 cm marking and precise time tracking in order to achieve the required velocities. Further, force was controlled. Stroking velocity and force were extensively trained by the experimenter prior to the experiment in performing video guided stroking with brush and hand on a fine tuned scale. In addition, force was controlled in the nonsocial conditions by checking the bending of the brushes hair, which was similar to the bending resulting a 0.4N force in a robot guided stroking (RTS). For the human condition, stroking force was additionally adjusted to match the brush force by feedback from the stroked participants in the pretest.

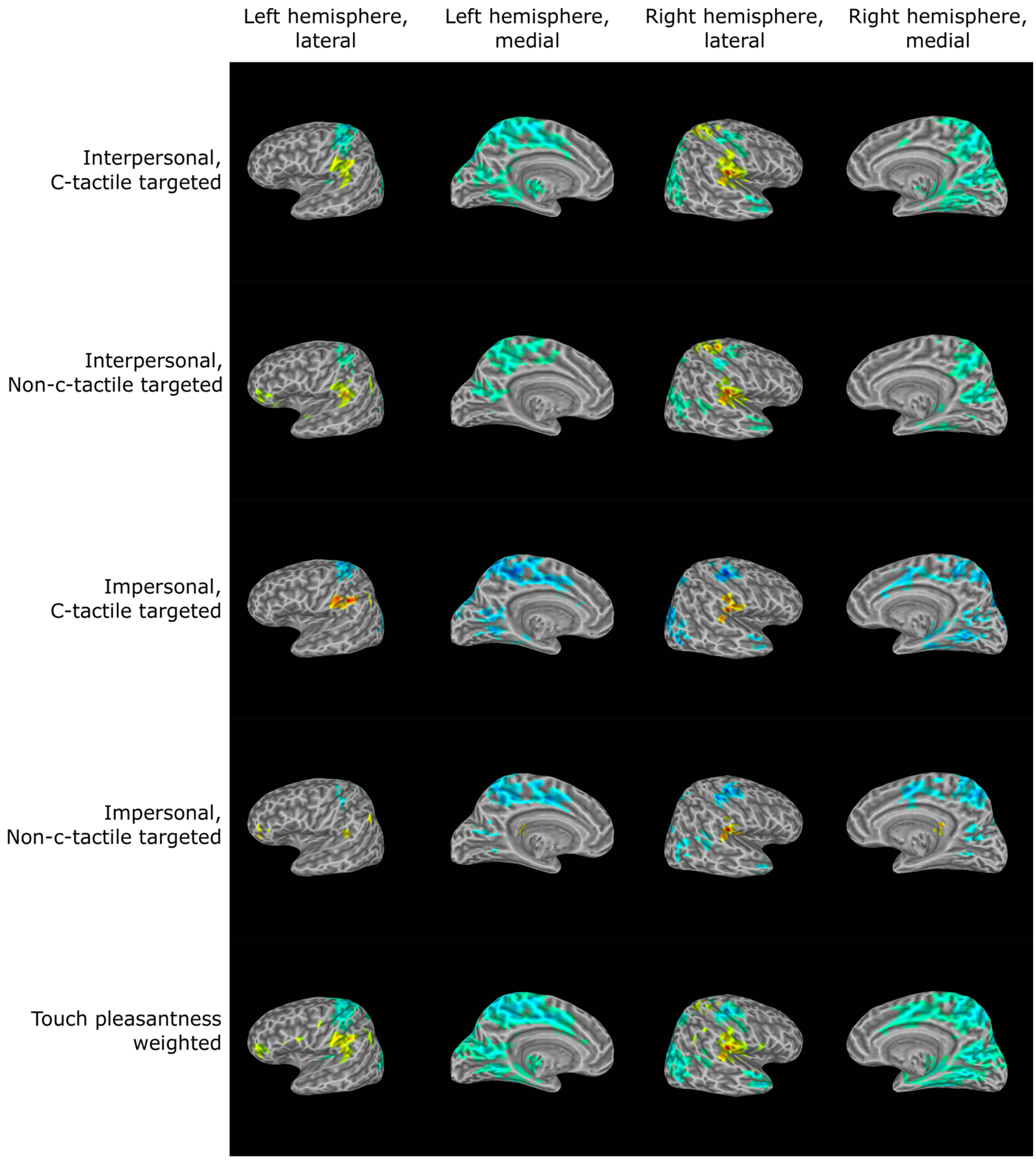

Supplementary Figure 1 – activation and deactivation in all four touch conditions and the "touch pleasantness weighted"-contrast. All of the four touch conditions led to a strong blood oxygen level dependent (BOLD) signal increase in the secondary somatosensory cortex which extended to the superior temporal gyrus and in the primary somatosensory cortex. These activations were more pronounced in the interpersonal compared to impersonal touch conditions. We further identified reduced BOLD response within the DMN in each of the four touch conditions. Please note: any colored region is suprathreshold corresponding to p<0.001, uncorrected and two-tailed. Hotter/cooler colors correspond to more extreme t-values.

Condition	Cluster size	T value	MNI coordinates x	y	z	FWE corrected p value: peak level
**Deactivation in touch conditions vs Baseline**						
**Interpersonal Touch, c-tactile targeted**						
L Superior Parietal Gyrus, Precentral Gyrus, Area 4a/5M/7a	675	8.55	-25	-20	76	<0.001
Right Precentral Gyrus, Area 1/3a/3b/4p	56	5.90	41	-18	58	0.002
R Superior Temporal Gyrus, Middle Temporal Gyrus	136	6.51	57	-6	-14	<0.001
**Interpersonal Touch, non-c-tactile targeted**						
R+L Area 5M, Sup. Parietal Lobe	173	7.82	15	-62	24	0.003
R Inferior Parietal Gyrus	145	6.16	15	-62	24	<0.001
R Precentral Gyrus + Postcentral Gyrus (Area1/2/3b/4p)	173	7.82	49	-22	58	<0.001
R Hippocampus	59	6.01	29	-22	-22	0.001
L Medial Cingulate Cortex	202	4.70	1	-44	44	0.008
**Impersonal Touch, c-tactile-targeted**						
L Superior Parietal Lobe, Precentral Gyrus, Paracentral Lobe	46	6.19	-27	-22	74	<0.001
R Precentral Gyrus	142	6.15	37	-18	54	<0.001
**Impersonal Touch, non-c-tactile-targeted**						
L Sup. Parietal Lobus, Postcentral Gyrus	35	6.43	-19	-28	80	<0.001
R Postcentral Gyrus	212	7.11	49	-22	60	<0.001
**Touch pleasantness weighted**						
L Sup. Parietal Lobe	4544	9.31	-25	-20	76	<0.001
R Sup. Parietal Lobe, Sup. Occipital gyrus	1911	7.53	27	-74	38	<0.001
R Pre- + Postcentral Gyrus	547	8.44	39	-18	56	<0.001
L Area hOc1+2	494	6.94	-15	-56	14	<0.001
L Superior Parietal Lobe, Sup. Occipital	820	7.28	-19	-86	40	<0.001

gyrus						
R Hippocampus	330	7.40	27	-32	-18	<0.001
R Superior Temporal Gyrus	294	7.39	55	-6	-16	<0.001
R Hippocampus	71	6.27	29	-30	-6	0.001
R Precentral Gyrus	22	6.00	17	-24	66	0.002

Supplementary Table S1 - neural deactivations vs Baseline of all touch conditions and the "touch pleasantness weighted" contrast. Data are extracted from a whole-brain analysis and only deactivations with peak-level FWE-corrected p-value<0.05 are reported.
